# Data on exopolysaccharides produced by *Bacillus* spp. from cassava pulp with antioxidant and antimicrobial properties

**DOI:** 10.1016/j.dib.2023.109474

**Published:** 2023-08-05

**Authors:** Thipphiya Karirat, Worachot Saengha, Sirirat Deeseenthum, Nyuk Ling Ma, Nantaporn Sutthi, Eakapol Wangkahart, Vijitra Luang-In

**Affiliations:** a Natural Antioxidant Innovation Research Unit, Department of Biotechnology, Faculty of Technology, Mahasarakham University, Khamriang, Kantarawichai, Maha Sarakham 44150, Thailand; b BIOSES Research Interest Group, Faculty of Science and Marine Environment, Universiti Malaysia Terengganu, 21030 Kuala Nerus, Terengganu, Malaysia; c Department of Agricultural Technology, Faculty of Technology, Mahasarakham University, Khamriang, Kantarawichai, Maha Sarakham 44150, Thailand; d Applied Animal and Aquatic Sciences Research Unit, Division of Fisheries, Faculty of Technology, Mahasarakham University, Khamriang, Kantarawichai, Maha Sarakham 44150, Thailand

**Keywords:** DPPH radical scavenging activity, Hydroxyl radical scavenging activity, Polysaccharide, Agricultural residues, *Streptococcus agalactiae*, *Staphylococccus aureus*, Agro-biowaste, Zero waste

## Abstract

This data evaluated the capacity of *Bacillus* spp. isolated from Thai milk kefir to produce exopolysaccharide (EPS) on cassava pulp and tested its antioxidant and antibacterial properties. Thailand's starch industry generates million tons of cassava pulp, which is underutilized or bio-transformed into higher-value bioproducts. Antioxidant and antibacterial bacterial exopolysaccharides are beneficial in the food, feed, pharmaceutical, and cosmetic industries. Moisture, ash, fat, protein, fiber, starch, sugar, neutral detergent fiber (NDF), acid detergent fiber (ADF), and acid detergent lignin (ADL) were analyzed from cassava pulp as an EPS substrate. After 3 days of bacterial fermentation, EPS generation, culture pH, reducing sugar amount, and bacterial count were recorded. Antioxidant activities and bioactive content including hydroxyl radical scavenging activity, 2,2-diphenyl-1-picrylhydrazyl (DPPH) radical scavenging activity, ferric reducing antioxidant power (FRAP), total phenolic and flavonoid content (TPC and TFC), and antimicrobial activity against two Nile tilapia pathogens (*Streptococcus agalactiae* and *Staphylococcus aureus*) from different *Bacillus* species were evaluated. Proximate analysis, dinitrosalicylic acid assay, pH value record, bacterial count using spread plate method, antioxidant activity and bioactive content assays via spectrophotometry, and agar disk diffusion were the main approaches. This study used microbial cell factories to convert agro-biowaste, such as cassava pulp, into EPS bioproducts which accords with a bio-circular green economy model.

**Specifications Table**

**Table:** 

Subject	Biological sciences
Specific subject area	Applied Microbiology; Biotechnology
Type of data	Table and figures
How the data were acquired	Proximate analysis, dinitrosalicylic acid (DNS) procedure, pH meter evaluation, total plate count by series of dilutions and spread plate methodology, spectrophotometry in antioxidant activity hydroxyl radical scavenging activity, 2,2-diphenyl-1-picrylhydrazyl (DPPH) radical scavenging activity, ferric reducing antioxidant power (FRAP), total phenolic and flavonoid content (TPC and TFC), and agar disk diffusion assay.
Data format	Raw and analyzed data
Description of data collection	Cassava pulp as EPS substrate was analyzed for moisture, ash, fat, protein, fiber, starch, sugar, neutral detergent fiber (NDF), acid detergent fiber (ADF), and acid detergent lignin (ADL). After 3-day fermentation, EPS production in fresh and dry weight, pH of culture, reducing sugar concentration, and bacterial numeration were reported. Antioxidant activities and bioactive content include hydroxyl radical scavenging, 2,2-diphenyl-1-picrylhydrazyl radical scavenging, ferric reducing antioxidant power, total phenolic and flavonoid content, and antimicrobial activity against Nile tilapia pathogens *S. agalactiae* and *S. aureus.*
Data source location	*Source of cassava pulp* *Institution: Korat Flour Industry Co., Ltd.* *City: Nakhon Ratchasima* *Country: Thailand* *Latitude and longitude for collected samples: latitude 14.8978033° N and longitude 102.0699811° E.* *Source of Bacillus* spp. *Institution: Natural Antioxidant Innovation Research Unit, Department of Biotechnology, Faculty of Technology, Mahasarakham University* *City/Town/Region: Khamriang, Kantarawichai, Maha Sarakham* *Country: Thailand* Latitude and longitude for collected samples: latitude 16.2440° N and longitude 103.2490° E.
Data accessibility	Raw dataset for bacterial exopolysaccharide production using cassava pulp as substrate by *Bacillus* spp. isolated from Thai milk kefir with its antioxidant and antimicrobial capacities, [11] https://data.mendeley.com/datasets/dt7cf566p9/2

## 1 Value of the Data

• This work presented the raw data obtained during the 3-day fermentation of EPS production using cassava pulp as substrate by eight different *Bacillus* spp. The data consisted of the EPS-producing content of each strain and its bioactivities.
• The dataset would provide insight into selecting the bacterial strain and using cassava pulp as substrate to generate a high EPS content with antioxidant activity and antimicrobial properties against two bacterial pathogens of Nile tilapia.
• The dataset is useful for agriculturists, researchers, and the private sector who wish to valorize cassava pulp via bacterial fermentation to produce bioactive EPS products.
• It is anticipated that the applicability of the dataset will allow for the commercialization of crude EPS bioproducts as feed additives.
• This work proposes an alternative, low-cost means of producing value-added EPS bioproducts from other agro-biowastes in addition to cassava pulp via bacterial fermentation, thereby enabling a bio-circular green economy.

## 2 Objective

This work aimed to determine the bioactivities of EPSs produced from cassava pulp (agro-industry-sourced biowaste) as substrate via a 3-day fermentation by different *Bacillus* spp.

## 3 Data Description

Chemical composition analysis of cassava pulp showed the predominance of starch and sugar at 64.12% followed by neutral detergent fiber (NDF) at 33.22%, acid detergent fiber (ADF) at 24.32%, fiber at 17.56% and protein at 12.09% (Table 1). The compositions of ash, fat, protein and acid detergent lignin (ADL) were all below 5%. This data is useful when it comes to considering the influence of chemical composition of substrate on EPS synthesis by *Bacillus* spp.

**Table 1:** Chemical composition analysis of cassava pulp.

Substrate	Moisture%	Ash%	Fat%	Protein%	Fiber%	Starch and sugar (%)(NFE)	% NDF	% ADF	% ADL
Cassava pulp	12.09 ± 0.10	3.33 ± 0.03	0.10 ± 0.02	2.84 ± 0.12	17.56 ± 0.30	64.12 ± 0.47	33.22 ± 0.14	24.32 ± 0.26	3.76 ± 0.30

%Nitrogen Free Extract (NFE) = 100 - [%Moisture +%Ash+%Protein +%Fat +%Fiber].

Neutral detergent fiber (NDF), Acid detergent fiber (ADF), Acid detergent lignin (ADL).

**Fig. 1:**
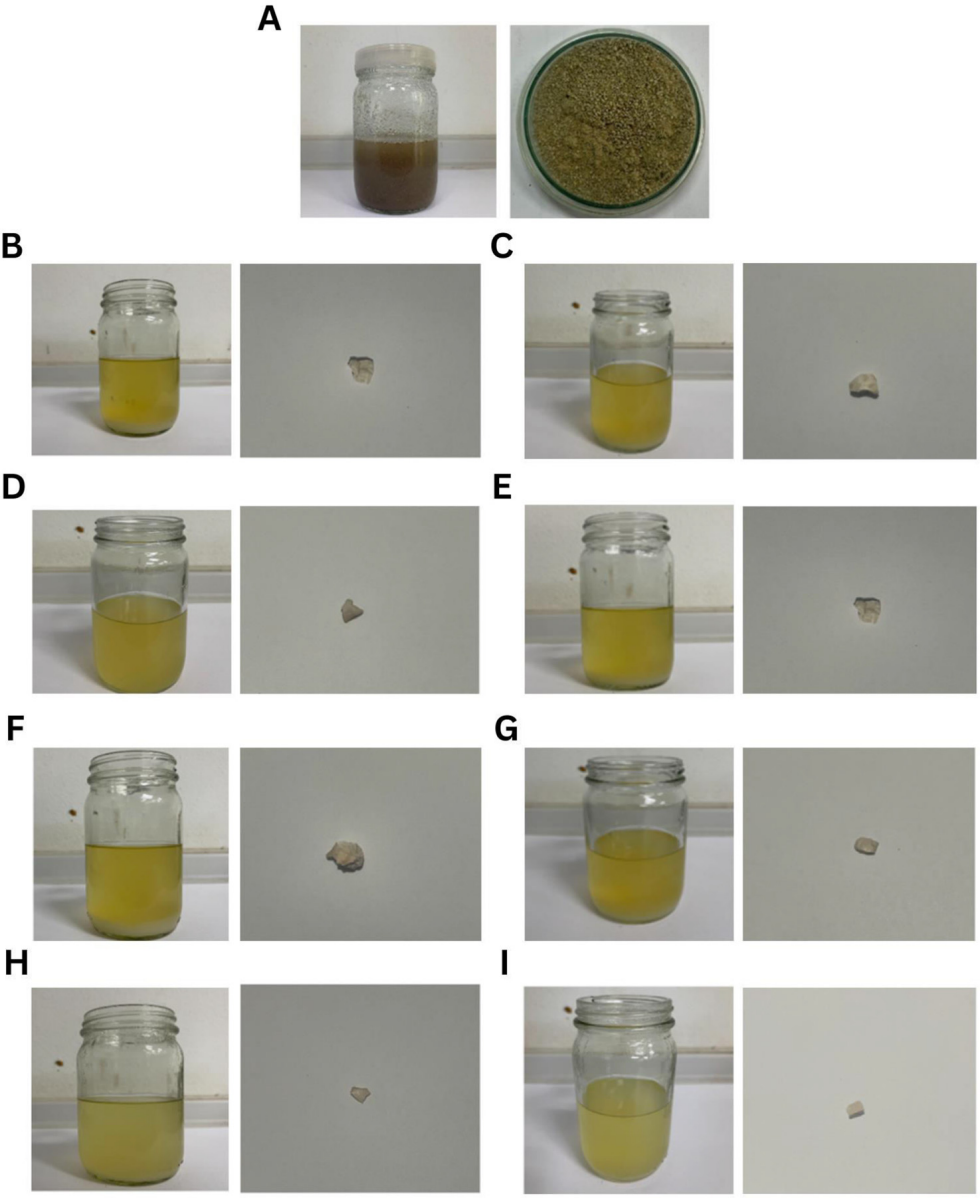
EPS production from cassava pulp by eight *Bacillus* bacteria over 3 days. (A) Cassava pulp substrate in the medium at day 0 (B) *B. amyloliquefaciens* KW1**,** (C) *B. amyloliquefaciens* KW8, (D) *B. amyloliquefaciens* KW9, (E) *B. amyloliquefaciens* KW10, (F) *B. tequilensis* PS21, (G) *B. teqilensis* PS22, (H) *B. teqilensis* PS23, and (I) *B. tequilensis* PS24.

Eight EPS-producing bacteria from the *Bacillus* genus isolated from Thai milk kefir were tested in this work [1]. Bacterial EPS production from cassava pulp by *Bacillus* spp. over 3 days was shown in Fig. 1. On day 0, the medium containing cassava pulp substrate appeared brownish (Fig. 1A)**;** however, over 3 days of bacterial fermentation, the culture appeared yellowish (Fig. 1B–1I). The crude EPS products from each strain looked similar, with whitish and creamy features**.**

Different *Bacillus* bacteria produced EPS differently. *B. tequilensis* PS21 produced the highest EPS content of 11.38 g FW/100 mL and 1.49 g DW/100 mL (Table 2). The pH values of the media in all strains dropped slightly from initially 7.0 (day 0) to about 6.31–6.44 over 3 days (Table 2). Similarly, the total plate count (TPC) of bacteria was similar (log 8.82–9.00 CFU/mL on day 3) in all strains.

**Table 2:** Bacterial EPS production using cassava pulp by *Bacillus* spp. over 3 days.

Bacteria	Fresh EPS (g FW/100 mL)	Dry EPS (g DW/100 mL)	pH day 1 day 2 day 3	Reducing sugar (mg/mL) day 1 day 2 day 3	TPC (log CFU/mL) day 1 day 2 day 3
*B. amyloliquefaciens* KW1	10.56 ± 0.45^a^	1.09 ± 0.16^a^	6.72 ± 0.15 6.25 ± 0.10 6.37 ± 0.05	0.548 ± 0.011 1.166 ± 0.009 0.993 ± 0.035	8.68 ± 0.08 8.82 ± 0.02 9.00 ± 0.01
*B. amyloliquefaciens* KW8	9.85 ± 0.50^b^	0.81 ± 0.16^b^	6.76 ± 0.07 6.61 ± 0.04 6.44 ± 0.05	0.550 ± 0.009 0.936 ± 0.016 0.847 ± 0.018	8.59 ± 0.09 8.71 ± 0.02 8.84 ± 0.03
*B. amyloliquefaciens* KW9	8.43 ± 0.69^c^	0.69 ± 0.05^c^	6.73 ± 0.04 6.54 ± 0.04 6.36 ± 0.04	0.580 ± 0.019 1.042 ± 0.005 0.745 ± 0.001	8.59 ± 0.05 8.75 ± 0.01 8.83 ± 0.03
*B. amyloliquefaciens* KW10	8.75 ± 0.52^c^	0.82 ± 0.12^b^	6.72 ± 0.11 6.55 ± 0.11 6.40 ± 0.06	0.559 ± 0.011 0.959 ± 0.001 0.693 ± 0.015	8.58 ± 0.02 8.74 ± 0.02 8.83 ± 0.02
*B. tequilensis* PS21	11.38 ± 1.00^a^	1.49 ± 0.23^a^	6.73 ± 0.08 6.59 ± 0.12 6.40 ± 0.12	0.635 ± 0.005 1.203 ± 0.039 1.011 ± 0.004	8.74 ± 0.03 8.86 ± 0.02 9.00 ± 0.01
*B. tequilensis* PS22	9.61 ± 0.33^b^	0.76 ± 0.07^b^	6.74 ± 0.09 6.53 ± 0.04 6.32 ± 0.03	0.593 ± 0.031 0.928 ± 0.010 0.713 ± 0.036	8.57 ± 0.03 8.72 ± 0.02 8.82 ± 0.01
*B. tequilensis* PS23	8.98 ± 0.32^b^	0.58 ± 0.13^c^	6.66 ± 0.11 6.49 ± 0.13 6.31 ± 0.09	0.585 ± 0.023 0.933 ± 0.011 0.734 ± 0.001	8.53 ± 0.02 8.71 ± 0.01 8.84 ± 0.01
*B. methylotrophicu*s PS24	9.28 ± 0.08^b^	0.59 ± 0.09^c^	6.80 ± 0.07 6.53 ± 0.06 6.33 ± 0.05	0.568 ± 0.018 1.074 ± 0.032 0.873 ± 0.003	8.54 ± 0.03 8.71 ± 0.02 8.82 ± 0.01

FW = Fresh Weight; DW = Dry Weight; TPC = Total Plate Count; CFU = Colony Forming Unit. Statistically significant differences (*p <* 0.05) are denoted by varying lowercase letters in the columns.

Antioxidant activities and bioactive contents of bacterial EPSs from cassava pulp are shown in Table 3. The dextran (20 mg/mL) EPS standard exhibited the most potent 2,2-diphenyl-1-picrylhydrazyl (DPPH) radical scavenging activity of 73.98%, a ferric reducing antioxidant power (FRAP) value of 2.23 mg Fe^2+^/g DW, and hydroxyl radical scavenging activity of 80.94%. Amongst eight strains, EPS produced by *B. tequilensis* PS21 displayed the strongest DPPH radical scavenging activity of 43.93%, a FRAP value of 0.71 mg Fe^2+^/g DW, and a hydroxyl radical scavenging activity of 44.26% (Table 3). Likewise, *B. tequilensis* PS21 displayed the highest TPC of 6.31 mg GAE/g DW.

**Table 3:** Antioxidant activities and bioactive contents of bacterial EPSs from cassava pulp.

	Antioxidant activities and bioactive contents				
EPS samples from bacteria	DPPH radical scavenging activity (%)	FRAP (mg Fe^2+^/g DW)	Hydroxyl radical scavenging activity (%)	TPC (mg GAE/g DW)	TFC (mg RE/g DW)
*B. amyloliquefaciens* KW1	43.47 ± 0.52^bc^	0.58 ± 0.01^cd^	22.25 ± 0.55^de^	2.65 ± 0.08^de^	2.97 ± 0.58^e^
*B. amyloliquefaciens* KW8	32.27 ± 0.49^d^	0.55 ± 0.02^de^	23.14 ± 0.25^cd^	2.09 ± 0.47^e^	3.51 ± 0.66^e^
*B. amyloliquefaciens* KW9	30.79 ± 0.62^f^	0.62 ± 0.10^cd^	22.81 ± 0.70^de^	4.18 ± 0.54^c^	5.14 ± 0.66^d^
*B. amyloliquefaciens* KW10	42.73 ± 0.65^c^	0.66 ± 0.03^bc^	20.03 ± 1.67^e^	3.27 ± 0.32^cd^	11.18 ± 0.40^b^
*B. tequilensis* PS21	43.93 ± 0.27^b^	0.71 ± 0.07^b^	44.26 ± 0.87^b^	6.31 ± 2.32^b^	7.85 ± 0.51^c^
*B. tequilensis* PS22	28.81 ± 0.42 g	0.47 ± 0.03^e^	24.71 ± 0.92^c^	1.14 ± 0.07^e^	3.21 ± 0.05^e^
*B. tequilensis* PS23	31.21 ± 0.72^e^	0.48 ± 0.05^e^	21.28 ± 0.74^de^	3.24 ± 0.36^cd^	9.05 ± 0.31^b^
*B. methylotrophicu*s PS24	28.78 ± 0.58 g	0.47 ± 0.01^e^	23.54 ± 0.55^cd^	3.28 ± 0.12^cd^	9.35 ± 0.56^b^
Dextran (20 mg/mL)	73.98 ± 0.27^a^	2.23 ± 0.04^a^	80.94 ± 0.46^a^	19.98 ± 0.44^a^	18.47 ± 1.16^a^

Statistically significant differences (*p <* 0.05) are denoted by varying lowercase letters in the columns. DPPH = 2,2-diphenyl-1-picrylhydrazyl; FRAP = ferric reducing antioxidant power; TPC = total phenolic content; TFC = total flavonoid content; GAE = gallic acid equivalent; RE = rutin equivalent.

**Fig. 2:**
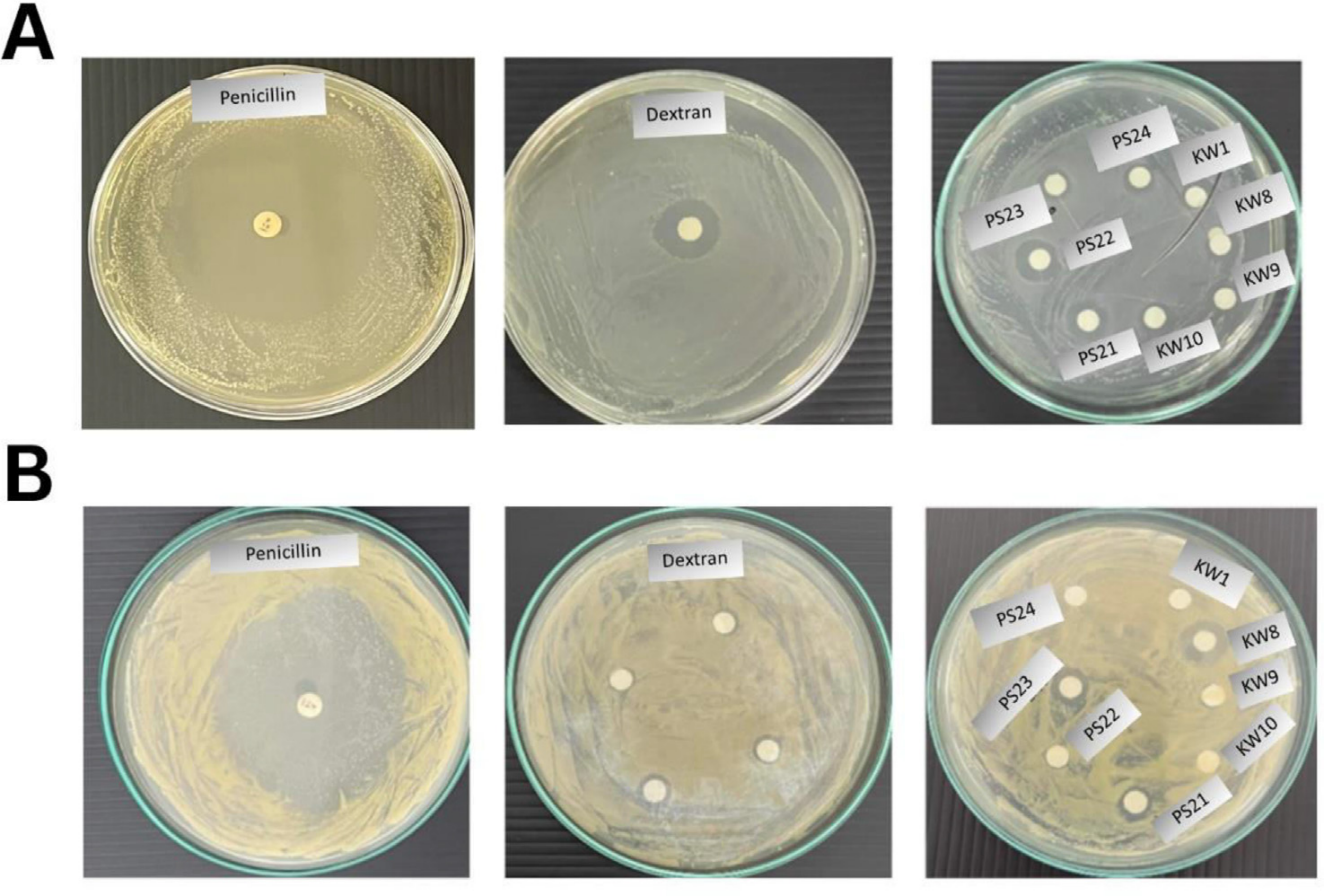
Antibacterial activity of bacterial EPSs against two pathogens of Nile tilapia by agar disk diffusion method. (A) *S. agalactiae* and (B) *S. aureus*. The first picture is penicillin positive control, the second picture is dextran EPS standard, and the third picture is eight bacterial EPSs from cassava pulp. Inhibition zones in mm diameter were measured.

Antibacterial activities of EPSs by eight *Bacillus* spp. on two common bacterial pathogens that cause infection in Nile tilapia, *S. agalactiae* and *S. aureus*, using an agar disk diffusion method are shown in Fig. 2 and Table 4.

**Table 4:** Antibacterial activity of eight bacterial EPSs against *S. agalactiae* and *S. aureus*.

	Inhibition zone (mm diameter)	
EPS samples from bacteria	*S. agalactiae*	*S. aureus*
*B. amyloliquefaciens* KW1	6.66 ± 0.35^e^	0.00 ± 0.00^g^
*B. amyloliquefaciens* KW8	7.36 ± 1.10^e^	7.50 ± 0.50^b^
*B. amyloliquefaciens* KW9	6.64 ± 0.77^e^	4.20 ± 0.10^f^
*B. amyloliquefaciens* KW10	6.29 ± 0.25^e^	0.00 ± 0.00^g^
*B. tequilensis* PS21	10.12 ± 0.54^d^	4.73 ± 0.25^e^
*B. tequilensis* PS22	12.12 ± 0.68^c^	4.12 ± 0.08^f^
*B. tequilensis* PS23	6.05 ± 0.57^f^	5.83 ± 0.76^d^
*B. methylotrophicu*s PS24	5.51 ± 0.23^g^	0.00 ± 0.00^g^
Penicillin (10 µg/mL)	64.16 ± 0.61^a^	50.23 ± 0.25^a^
Dextran (20 mg/mL)	14.83 ± 0.29^b^	6.18 ± 0.06^c^

Statistically significant differences (*p* < 0.05) are denoted by varying lowercase letters in the columns.

Penicillin antibiotics (10 µg/mL disk) as a positive control showed the highest clear zones on both pathogens, followed by dextran (20 mg/mL) as an EPS standard against *S. agalactiae* (Table 4 and Fig. 2A). However, dextran was not that effective against *S. aureus* (Fig. 2B).

For *S. agalactiae, B. tequilensis* PS22 displayed the most potent microbial activity, followed by *B. tequilensis* PS21**,** and *B. amyloliquefaciens* KW8 and KW9 (Table 4). For *S. aureus, B. amyloliquefaciens* KW8, *B. tequilensis* PS23**,** and *B. tequilensis* PS21 are the best three strains for antimicrobial activity. However, three strains of *B. amyloliquefaciens* KW1**,** *B. amyloliquefaciens* KW10 and *B. methylotrophicu*s PS24 did not exhibit antimicrobial activity towards *S. aureus* at all.

## 4 Experimental Design, Materials and Methods

### 4.1 Cassava Pulp Substrate Preparation

The cassava pulp was ground into a powder by milling and sieving, and the resulting powder was stored in a desiccator until it was required [2].

### 4.2 Chemical Composition Analysis

Proximate analysis according to standard AOAC procedures [3] were used to determine the levels of ash, moisture, and crude fat. The percentages of neutral detergent fiber (NDF), acid detergent fiber (ADF), acid detergent lignin (ADL) were calculated in the same manner as previously published [4]. The amount of crude protein was determined using the Kjeldhal technique.

### 4.3 Bacterial Sources

Eight EPS-producing bacteria from the *Bacillus* genus isolated from Thai milk kefir of Kamphaeng Phet Province, Thailand [1] were used for EPS production using cassava pulp as substrate.

### 4.4 Cultivation of Bacterial Strains

*Bacillus* spp. were successfully grown in Tryptic Soy Broth (TSB)(Oxoid, Basingstoke, UK) pH 7.0 (17 g/L tryptone, 3 g/L phytone, 5 g/L NaCl, 2.5 g/L glucose). All bacteria were cultured in an aerobic environment for 24 h at 37 °C, 150 rpm in a shaking incubator (LSI-1005R, Daihan LabTech, Kyonggi-do, South Korea). The OD_600nm_ was measured using M965+ microplate reader (MeterTech, Taipei, Taiwan) and adjusted to 0.1 for bacterial suspension before being inoculated into 5% (w/v) cassava pulp media.

### 4.5 Preparation of Cassava Pulp for Bacterial EPS Production

Cassava pulp powder at 5% (w/v) was mixed in autoclaved 100 mL distilled water. This was used as the medium for bacterial EPS production. The inoculum of overnight bacterial culture at 3% (v/v) was inoculated into the media and aerobically incubated for 3 days at 37 °C with agitation at 150 rpm. The reducing sugar content and pH values were recorded daily for 3 days using the DNS method [5] and a pH meter (FiveEasy Plus pH meter FP20, Port Melbourne, Australia). Glucose standard and DNS were purchased from Sigma-Aldrich, MO, USA. Total plate count (TPC) for bacterial growth at day 1–3 was conducted using the spread plate technique and serial dilutions. EPS quantity was determined on day 3.

### 4.6 Extraction of Crude EPS

After 3 days, the bacterial culture was spun at 16,100 g at 4 °C for 30 min in a centrifuge (Hettich® Universal 320/320R centrifuge, Kirchlengern, Germany). Two hundred milliliters of cold absolute ethanol (RCI Labscan Limited, Bangkok, Thailand) were mixed into the supernatant at 4 °C for 24 h without shaking. After centrifugation, EPS precipitate was rinsed twice with 100% ethanol. Fresh EPS was weighed using a fine balance (Presica 25A, Switzerland) and dried crude EPS was obtained as previously described [6]. Next, dried crude EPS (20 mg) was resuspended in 1 mL of sterile distilled water, filtered through 0.20 µm membrane filter (Sartorius Minisart™ High Flow Syringe Filters, Thermo Fisher Scientific Inc., MA, USA) and stored at −20 °C until use.

### 4.7 Antioxidant Activity and Bioactive Contents

#### 4.7.1 Hydroxyl Radical Scavenging Activity

The ability of EPSs to absorb hydroxyl radicals was determined [7]. In order to initiate the reaction, 0.5 mL of EPS (20 mg/mL), 0.5 mL of FeSO_4_ (0.8 mM), 0.5 mL of H_2_O_2_ (0.01% v/v), 1 mL of sodium phosphate buffer (0.2 M, pH 7.3), and 0.5 mL of 1,10-phenanthroline (0.8 mM) were mixed to react. The containers were left for 30 min at 37 °C. At 536 nm, the absorbance of compounds was measured using M965+ microplate reader (MeterTech, Taipei, Taiwan).

$$\text{Scavenging}\;\text{activity}\;\left(\%\right)\;=\;\left[1-\left(A_{\text{sample}}-A_{\text{blank}}\right)/A_{\text{control}}\right]\;\times \;100$$

where A_blank_ contained deionized water, A_control_ contained all reagents without the EPS sample, and A_sample_ contained all reagents and EPS sample. Dextran was used as an EPS standard. All chemicals were from Sigma-Aldrich, MO, USA.

#### 4.7.2 DPPH Radical Scavenging Activity

In a test tube, 2.0 mL of water, 1 mL of EPS sample (20 mg/mL), and 0.4 mL of DPPH ethanolic solution (0.5 mM) were mixed [6]. Thirty minutes were spent incubating the containers at 30 ± 3 °C in the dark. At 517 nm, the absorbance was recorded using M965+ microplate reader (MeterTech, Taipei, Taiwan).

$$\text{Scavenging}\;\text{activity}\;\left(\%\right)\;=\;\left[1-\left(A_{\text{sample}}-A_{\text{blank}}\right)/A_{\text{control}}\right]\;\times \;100$$

where A_blank_ contained deionized water, A_control_ contained all reagents without the EPS sample, and A_sample_ contained all reagents and EPS sample. Dextran was used as an EPS standard. All chemicals were from Sigma-Aldrich, MO, USA.

#### 4.7.3 FRAP

EPS (20 µL of a 20 mg/mL stock solution) was treated with a FRAP reagent (180 µL) comprised of 20 mM FeCl_3_, 10 mM 2,4,6-Tri (2-pyridyl) s-triazine, and a 0.3 M acetate buffer at pH 3.6, as previously reported [8]. The microplate reader recorded 593 nm absorption in triplicate after 30 min. Ferrous II sulfate was used as a standard. All chemicals were from Sigma-Aldrich, MO, USA.

#### 4.7.4 TPC

This was analyzed in the same way as was explained in the previous work [9]. A total volume of 100 µL of 10% Folin-Ciocalteu solution was mixed with 20 µL of EPS (20 mg/mL), 80 µL of 7.35% sodium carbonate. After a 30-min dark response, a record at A_765nm_ was taken using M965+ microplate reader (MeterTech, Taipei, Taiwan). Gallic acid served as a standard. All chemicals were from Sigma-Aldrich, MO, USA.

#### 4.7.5 TFC

EPS (20 mg/mL) was mixed with deionized water (60 µL), 10% aluminum trichloride (10 µL), and 5% sodium nitrate (10 µL) using a micropipette. After reacting for 30 min, A_420nm_ was recorded after 100 µL of 1 M NaOH addition. A standard of rutin was employed. All chemicals were from Sigma-Aldrich, MO, USA.

### 4.8 Antimicrobial Activity

An agar disk diffusion test was carried out. *S. agalactiae* and *S. aureus*, the most common infectious agents in Nile tilapia, were employed to test EPSs' antibacterial efficacy. *S. agalactiae* EW1 (GenBank accession no. OR272051.1) was isolated from diseased Nile tilapia in the northeastern region of Thailand [10]. *S. aureus* TISTR 517 (ATCC 25923) (GenBank accession no. OP522324.1) was obtained from Thailand Institute of Scientific and Technological Research (TISTR), Thailand. Both bacteria were grown for 24 h at 37 °C in Luria-Bertani (LB) broth (HiMedia, Maharashtra, India), and the cultures were adjusted to 10^8^ CFU/mL determined by A_600nm_. Paper discs (4 mm diameter) were placed upon LB agar plates inoculated with 100µL of bacterial suspension. Each sample disk contained 20 µL of sterilized EPS (20 mg/mL). LB agar dishes were incubated at 37 °C for 48 h. The antimicrobial activity was assessed as the record of the inhibition zone diameter in mm. Dextran (20 mg/mL) was used as an EPS standard, and penicillin (10 µg/mL) from Sigma-Aldrich, MO, USA was utilized as a positive control.

### 4.9 Statistical Analysis

Triplicates were used for each treatment, and mean ± SD was reported. SPSS Demon version (IBM, Armonk, NY, USA) was used to assess significant mean differences using ANOVA and Duncan's multiple range test at *p <* 0.05.

## Ethics Statements

This article does not contain any dataset involving animals or human participants performed by any of the authors.

## CRediT authorship contribution statement

**Thipphiya Karirat:** Methodology, Formal analysis. **Worachot Saengha:** Data curation. **Sirirat Deeseenthum:** Investigation, Resources. **Nyuk Ling Ma:** Supervision, Writing – review & editing. **Nantaporn Sutthi:** Methodology, Resources. **Eakapol Wangkahart:** Methodology, Resources. **Vijitra Luang-In:** Conceptualization, Funding acquisition, Writing – original draft, Writing – review & editing.

## Declaration of Competing Interest

The authors declare that they have no known competing financial interests or personal relationships that could have appeared to influence the work reported in this paper.

## Data Availability

Raw dataset for bacterial exopolysaccharide production using cassava pulp as substrate by Bacillus spp. isolated from Thai milk kefir with its antioxidant and antimicrobial capacities (Original data) (Mendeley Data).
